# The effect of intravenous iron on postoperative transfusion requirements in hip fracture patients: study protocol for a randomized controlled trial

**DOI:** 10.1186/1745-6215-14-288

**Published:** 2013-09-09

**Authors:** Martin Rowlands, Daren P Forward, Opinder Sahota, Iain K Moppett

**Affiliations:** 1 Division of Anaesthesia and Intensive Care, University of Nottingham, Nottingham, UK; 2 Department of Trauma and Orthopaedics, Nottingham University Hospitals NHS Trust, Nottingham NG7 2UH, UK; 3 Department of Healthcare of Older People, Nottingham University Hospitals NHS Trust, Nottingham NG7 2UH, UK

**Keywords:** Aged 70 and over, Anaemia: iron deficiency, Anaemia: therapy, Ferric compounds: administration and dosage, Ferric compounds: therapeutic use, Hip fractures: surgery, Human beings, Perioperative period, Postoperative complications: drug therapy

## Abstract

**Background:**

Anaemia following hip fracture is common. Approximately 30 to 45% of patients have haemoglobin concentrations below population norms on admission, and around 10% are severely anaemic. Anaemia on admission, and in the postoperative period, is associated with poor outcomes with regard to mobility, postoperative mortality and readmission. There is currently no clear consensus on the optimal method of managing perioperative anaemia in this group of frail patients with frequent comorbidity. Liberal red cell transfusion in the postoperative period does not appear to improve outcome, whereas tranexamic acid appears to reduce transfusion rate at the expense of increased cardiovascular morbidity. There are encouraging results from one centre with the use of agents to stimulate red cell production, including intravenous iron and erythropoietin. UK practice differs significantly from these patients and these studies, and it is not clear whether these promising results will translate to the UK population.

**Methods/Design:**

This is a single-centre randomized controlled parallel group trial, in a British university hospital.Randomization is achieved using a website and computer-generated concealed tables. Participants are 80 patients 70 years or over with acute hip fracture undergoing operative repair. The intervention group receive three daily infusions of 200 mg iron sucrose, starting within 24 hours of admission. The control group receive standard hospital care at the discretion of the clinical team. Red cell transfusions for each group are given in accordance with standard clinical triggers. The primary outcome is an increase in mean reticulocyte count in the intervention group at day 7. Secondary outcome measures include haemoglobin concentrations, early and late transfusion rates, infectious and cardiovascular complications, mobility and 30-day mortality.

**Discussion:**

This is a pilot study to demonstrate haematopoietic efficacy of intravenous iron in this setting. Hence, we have chosen to measure change in reticulocyte count rather than the more clinically relevant differences in haemoglobin concentration or transfusion rate. If our results are positive, the study will provide the necessary information for development of a full-scale trial of intravenous iron.

**Trial registration:**

Current Controlled Trials ISRCTN76424792; UK Medicines and Healthcare products Regulatory Authority (EuDRACT: 2011-003233-34).

## Background

Anaemia following hip fracture is common. Approximately 30 to 45% of patients have haemoglobin concentrations ([Hb]) below population norms on admission and around 10% are severely anaemic. The average fall in [Hb] between admission and postoperative nadir is around 2 g/dl. Various authors [1-4] have found [Hb] on admission to be an independent risk factor for early [1,2] and late [3] postoperative mortality. Foss and colleagues [5] found postoperative [Hb] < 10 g/dl to be an independent risk factor for poor mobility in previously mobile patients, whilst Lawrence and colleagues [6] found that walking distance was strongly correlated with [Hb] > 10 g/dl. In healthy older people, mobility is better in individuals with [Hb] > 12 g/dl [7]. Health outcomes in the general older population are worse in the presence of anaemia [8].

Anaemia in the older hip fracture population is multifactorial. Ageing per se does not appear to cause anaemia [9], but ageing may be associated with reduced haematopoietic reserve, making anaemia more likely in the face of nutrient deficiency or blood loss [10]. In the community, 20% of women aged 80 to 85 are anaemic, with anaemia of chronic disease being the most common cause. These patients represent a major proportion of the hip fracture population. However, 30 to 45% of hip fracture patients are anaemic on admission [11], which may reflect frailer patients having poorer nutrition, being more likely to sustain hip fractures and subsequently having their [Hb] fall further, owing to acute fracture-related blood loss.

Pre-existing anaemia on admission is further exacerbated by the effects of intravenous rehydration, and small but significant intra-operative blood loss results in between one-third and two-thirds of patients receiving blood transfusions within the first few days following operation [5]. The likelihood of transfusion is related to fracture type: subtrochanteric and trochanteric fractures have the highest transfusion rates in both anaemic and nonanaemic subjects. Poor nutrition in the postoperative period is also unlikely to replenish iron stores. Surgery itself may further induce a functional iron deficiency due to humoral suppression of erythropoiesis [12]. Functional iron deficiency does not respond well to oral iron, as iron stores may be normal, and this suppresses intestinal absorption of iron. Indeed, postoperative oral iron has been shown to be of no benefit and also has a high incidence of side effects [13]. Whereas blood transfusion improves [Hb], some authors have found an association between transfusion and improved mobility [5]; others have not found this association, but found that transfusion might reduce readmission rates [14]. Infection rates may also be increased with the use of blood transfusion [15] and there are significant financial constraints with the current cost of a unit of blood in the UK around £200. A recently reported North American trial assessed the effects of liberal versus restrictive postoperative transfusion strategies following hip fracture in patients with cardiovascular risk factors [16-18]. That study found no differences in self-reported mobility or death at 60 days, though there was an increase in cardiovascular complications in the restrictive group. It would seem therefore, at this time that anaemia is a poor prognostic factor but that red cell transfusion does not necessarily alter outcome.

Alternatives to blood transfusion include reduction in blood loss in the perioperative period; stimulation of erythropoiesis with erythropoietin; and oral or intravenous iron.

Pharmacological reduction of blood loss with tranexamic acid has been assessed recently [19]. Although there was a trend to reduced transfusion requirements and reduced postoperative infections, there was also a trend towards increased thrombotic events in the tranexamic acid group and it is therefore not a recommended therapy in this group, despite being advocated for patients with multiple traumas [20].

Erythropoietin has been used in older people to stimulate erythropoiesis. However, the side effects of erythropoietin (hypertension, risks of thrombotic events) make it unattractive in the hip fracture population and the cost of erythropoietin used in these studies is high, over £200 per patient.

Intravenous iron is an attractive therapy in this population. Intravenous iron bypasses the intestinal barrier to absorption [21] that may be seen in functional iron deficiency and stimulates erythropoiesis. The effects of i.v. iron are believed to last for around 7 days, which covers the at-risk period for patients with hip fracture. In post-partum women, who commonly have moderate blood loss during delivery, i.v. iron has been shown to have a faster effect on [Hb] than oral iron [22]. Intravenous iron is believed to be much less likely to cause adverse reactions than in the past, following changes in its pharmaceutical preparation. Iron sucrose is associated with the lowest rate of life-threatening adverse events of all the i.v. iron preparations and these are considerably lower than transfusion-related severe side effects (0.11/million versus 10/million) [23]. Four studies from a Spanish group have reported the effects of i.v. iron on transfusion requirements, mortality and outcome compared with historical controls [21]. Cuenca and colleagues [24] reported a nonsignificant reduction in transfusion rate in patients with trochanteric fractures (55% vs. 44%). A prespecified subgroup of patients with [Hb] >12 demonstrated a significant reduction in transfusion rate (41% vs. 26%). In another retrospective study, transfusion rates were halved (37% vs. 15%) as were the number of units transfused, length of acute hospital stay and 30 day mortality [21]. In a parallel group study [25], transfusion rates were also more than halved (70% vs. 24%) as were the number of units transfused and postoperative infection rates. Recently, the same group have reported on a randomized controlled trial of intravenous iron therapy [26]. No difference was found in transfusion rates for the trial as a whole, but prespecified subgroup analysis demonstrated a reduction in transfusion requirements for patients with subcapital fractures and those admitted with [Hb] > 12 g/dl. Overall there is a reduction in both red cell transfusion and postoperative infection of around 40%; 30-day mortality and length of stay do not appear to be altered. Laboratory data support the positive effects of i.v. iron on erythropoiesis with statistically and clinically significant increases in reticulocyte counts and iron indices. These data used non-randomized comparison groups, except for the final study. Furthermore, clinical management is different from current UK practice and guidelines, with an average time to theatre of over 4 days, compared with a UK median time of around 48 hours [27]. Recent changes to UK tariffs strongly encourage operation within 36 hours [28,29].

Intravenous iron therapy is relatively inexpensive; 200 mg of iron sucrose costs around £15. Administration costs for i.v. iron are likely to be similar to blood transfusion, in terms of consumables and nursing time. Even if the only benefit of intravenous iron is to reduce transfusion rates, the potential cost savings are considerable. Potential benefits, such as a reduction in infectious complications, which are suggested by the current literature, would result in significant cost savings to the healthcare economy, as well as improved quality of care. The direct consumable costs are probably slightly in favour of i.v. iron. With an estimated i.v. iron cost of £43 and blood unit cost of £200, assuming that an average of two units are transfused when given, if i.v. iron reduces transfusion rates from 38% to 25%, the direct cost saving in transfusion-related costs is around £10 per patient. Unlike cost savings from length of stay, these are direct savings to the healthcare system that can be realised, as well as releasing a scarce resource for use elsewhere.

The current literature supports the hypothesis that i.v. iron might be an effective and safe treatment, reducing the need for blood transfusion following hip fracture. Given the differences between UK and Spanish hip fracture management, particularly regarding time to operation, it is unclear whether the promising results from the Spanish experience will be applicable to the UK hip fracture population. It is unknown at present whether i.v. iron would have sufficient erythropoietic effect in hip fracture patients to reduce transfusion rates in the early postoperative period.

A large randomized controlled trial of preoperative i.v. iron for patients with hip fracture may be appropriate. To inform appropriate design of such a trial, a pilot study evaluating the haematological effect of i.v. iron in this population is required.

## Methods/Design

### Study objectives

#### ***Primary aims***

To investigate whether intravenous iron given as three 200 mg doses over three days in patients with hip fracture causes an increase in reticulocyte count in the early postoperative period;

#### ***Secondary aims***

To discover whether intravenous iron has beneficial effects on other measures of haematopoiesis, transfusion requirements and patient outcomes.

#### ***Haematological measures***

As haematopoietic indices, full blood count and serum transferrin receptor concentrations will be measured. These will be collected daily for 7 days following admission. In addition, the number of patients requiring blood transfusion during hospital admission and the transfusion index (units/patient) will be determined.

#### ***Patient outcome measures***

A number of patient outcomes will be measured: postoperative infectious complications, cardiovascular complications, and the length of acute hospital stay. In addition functional mobility at days 1 to 3 will be assessed using a cumulated ambulation score. Transfusion-related costs (consumables and nursing time) will be determined, and overall acute healthcare costs will be estimated from length of stay, investigations and drug costs.

### Study design

This is a prospective, single-centre, randomized, parallel group controlled trial conducted at the Queen’s Medical Centre campus of Nottingham University Hospitals, Nottingham, UK. Study recruitment commenced in July 2012 when the first patient was randomized. Recruitment is expected to take 24 months.

### Randomization and blinding

Randomization (on a one-to-one allocation basis) is via a password-protected web-based randomization service provided by the Clinical Trials Support Unit and the sequence is not revealed until datalock. Participants are randomized within 24 hours of admission, either before or after surgery.

The treating team, research nurse collecting data and research team analyzing data will be blinded to treatment allocation. The laboratory will be blinded to treatment allocation. The patient and ward nursing staff will not be blinded, for practical reasons (Venofer is a dark brown, aqueous solution). There is no placebo.

Postoperative data collection is by a research nurse blinded to treatment allocation. The decision that a patient is medically fit for discharge is made by the multiprofessional team, when all are satisfied that the participant has no ongoing needs for acute hospital care. This team is blinded to participant allocation.

### Selection and withdrawal of participants

#### ***Recruitment***

Participants are identified by their presence on surgical lists and are recruited from the trauma wards. The investigator informs the participant or the participant’s nominated representative (other individual or other body with appropriate jurisdiction), of all aspects pertaining to participation in the study. Active participation in the study is for the first seven days of hospital admission. The study intervention is complete after the third day (administration of i.v. iron) with daily blood tests until day 7. The medical notes are reviewed following hospital discharge for in-hospital complications and medication use.

Participants are informed that entry into the trial is voluntary and that they are free to withdraw at any time without effect on subsequent care. Data on time to discharge and postoperative mortality are collected routinely and separately from this study. These outcome data are therefore available for all randomized participants.

Patients who are unable to provide consent for themselves are not included in this study, owing to its pilot nature and the off-label use of intravenous iron.

Patients for whom language may be a barrier are eligible for inclusion to the study using standard interpreting services. In practice, this is a very small proportion of the total older population in the study centre.

### Inclusion criteria

Patients listed for or having undergone surgical repair of fractured neck of femur, who are aged 70 years or over and are admitted directly to the study centre through the emergency department will be included.

### Exclusion criteria

Participants will be excluded if they have had an undisplaced intracapsular fracture (as this requires a very low transfusion rate), or if there are any of the following contra-indications to intravenous iron therapy:

• Severe infection on admission.

• Liver disease.

• Known sensitivity to intravenous iron.

Participants taking iron orally will be excluded, as the simultaneous use of oral iron is prohibited in accordance with the licensing of Venofer (although taking oral iron prior to admission is not).

Participants will also be excluded if they are currently taking clopidogrel or ticlopidine (local practice is not to delay surgery for patients taking clopidogrel or ticlopidine, but they are at increased risk of bleeding in the intra-operative and perioperative period); aspirin is not an exclusion medication. (UK doses (75 mg or 150 mg) are not associated with increased surgical bleeding).

### Informed consent

The capacity for providing consent is assessed routinely by the orthopaedic team, who decide whether the patient is competent to provide consent for the surgical procedure. If the orthopaedic team deem the patient unable to consent to their surgery, then the patient will be deemed incapable of consenting to enter the study. A member of the research team also performs an additional check of the participant’s ability to provide consent, immediately prior to starting the study. All members of the research team are trained at obtaining consent in accordance with guidance for good clinical practice [30].

### Study intervention

#### ***Investigation medical product administration***

Patients allocated to the investigation medical product will receive 200 mg iron sucrose (Venofer) on three separate occasions. T1 will be day 1 (within 24 hours of admission); T2 will be day 1 after the operation, or the second morning following admission if the patient has not yet gone to theatre; T3 will be day 2 after the operation, or the third morning following admission if the patient has not yet gone to theatre.

Administration of intravenous iron will be in accordance with manufacturer’s recommendations. 10 ml Venofer (200 mg iron) will be diluted in 100 mls 0.9% sodium chloride for injections (to give a final concentration of 2 mg/ml).

On the first occasion, the first 25 mg of iron (i.e. 12.5 ml of solution) will be infused as a test dose over a period of 15 minutes. If no adverse reactions occur during this time then the remaining portion of the infusion will be given at an infusion rate of 100 mls/hour.

Oral iron is prohibited for at least 5 days following administration of intravenous iron. Given the negative results of a recent study of oral iron in hip fracture [13], oral iron will not be permitted for any study participants until day 10.

#### ***Concomitant medication***

Analgesia will be provided in accordance with normal practice at the study centre. Nonsteroidal anti-inflammatory drugs are not prescribed routinely to this group of patients, and will be prohibited for patients in the study. Other medication will be prescribed by the attending medical staff as appropriate for each individual.

#### ***Concomitant treatments***

Physiotherapy, surgery and anaesthesia will be administered in accordance with normal clinical practice during the trial.

#### ***Laboratory analyses***

Blood samples will be taken from the participants on admission, and once each on days 1 to 7 following admission. Where possible, these blood samples will be taken at the same time as clinically required samples, to reduce the participants’ discomfort. Analysis will be carried out in the hospital’s haematology laboratory using standard techniques. This laboratory is certified in accordance with National Health Service standards, and undergoes its own quality assurance programme. Samples will be destroyed in accordance with normal procedures once analyzed, in accordance with the UK Human Tissue Act, 2006.

### Standard care

Standard care will be identical in both groups; only the administration of interventional medical product will differ.

All patients are admitted to dedicated trauma wards and cared for in accordance with UK ‘Best Practice Tariff’ [29]. This includes assessment by orthogeriatricians; operation within 36 hours of admission; and assessment of bone health and falls. All patients are cared for under a hip fracture care pathway, which involves rapid assessment and admission from the emergency department; intravenous crystalloid infusions from the time of admission and multiprofessional care and discharge planning. Operations are performed in dedicated trauma theatres by consultants in anaesthesia and orthopaedic trauma or senior trainees. The Queen’s Medical Centre has undertaken continuous, systematic audit of its hip fracture care since 2011 [4,11,31].

### Transfusion triggers

Perioperative transfusion will be at the discretion of the attending anaesthetist. Transfusion following return to the ward will be at the discretion of the ward team. Indications for transfusion are, in order of clinical priority:

• Ongoing blood loss with hypovolaemia;

• Symptomatic anaemia: anaemia with haemoglobin concentration < 10 g/dl associated with persistent hypotension, angina, heart failure or cerebral dysfunction;

• Asymptomatic anaemia with haemoglobin concentration < 8 g/dl;

• Relative anaemia ([Hb] significantly less than normal for the patient) and poor functional recovery.

### Cost analysis

Data on the cost of treatment will be calculated from drug use, investigations (diagnostic imaging, electrocardiography, blood testing) and the clinical record. This includes standardized costs for physiotherapy, occupational therapy and discharge planning. The cost of additional monitoring required for each patient will also be taken into consideration. Data will be presented in terms of total nonoperative costs, costs per day and excess costs attributable to treatment group.

### Statistics

Data will be analyzed by the research team in conjunction with a medical statistician, using the latest version of SPSS software. There will be no interim analysis.

The primary outcome measure, difference in mean reticulocyte count between groups in the full analysis set will be analyzed using unpaired *t* tests or Mann-Witney tests as appropriate. Differences between groups in maximal change in [Hb], serum transferrin concentration, postoperative mobility, number of units transfused and estimated costs will similarly be analyzed.

Risk ratios will be described for categorical data: the number of patients in each group with cardiovascular or infective complications; and the number of patients requiring transfusion. Descriptive statistics will be used to summarise length of stay and 30-day mortality.

Prespecified subgroup analysis will be undertaken according to admission haemoglobin concentrations ([Hb] <12 or >12 g/dl) and fracture type (sub-trochanteric, extracapsular, intracapsular, pathological).

In addition to the calculated values, confidence intervals and odds- ratios will be presented when appropriate. All clinical information including all adverse events will be presented in full. All secondary analyses will be interpreted with caution as the sample size calculation is based on the primary outcomes only. However, the level of power associated with secondary results will be investigated.

### Sample size and justification

We propose to study a convenient sample size, with 40 participants in each group. Groups of 35 participants each would provide a statistical power of 80% for finding an absolute difference in maximum reticulocyte count of 1%, assuming a standard deviation of approximately 1.6%. This would also provide a statistical power of 80% for finding a difference in serum transferrin receptor concentration of 0.33 mg/dl. This is a conservative estimate of effect size; Garcia-Erce et al. [25] found differences of 1.75% and 0.4 mg/dl respectively. The extra ten patients are included to account for drop-outs.

### Definition of datasets analyzed

The safety set will comprise all randomized participants who receive at least one dose of the study drug or are randomized to standard care. The full analysis set will comprise all randomized participants for whom at least initial and day 7 reticulocyte counts are available. The per-protocol set will comprise all participants in the full analysis set who are deemed to have no major protocol violations that could interfere with the objectives of the study. Safety summaries will be performed on the safety set.

### Reporting of adverse events

All adverse events will be recorded and closely monitored until resolution or stabilisation, or until it has been shown that the study treatment is not the cause. Participants will be asked to contact the study site immediately in the event of any serious adverse event. The chief investigator shall be informed immediately of any serious adverse events and shall determine seriousness and causality in conjunction with any treating medical practitioners.

All treatment-related serious adverse events will be recorded and reported to the research ethics committee as part of the annual reports. Unexpected serious adverse events will be reported to the research ethics committee and the sponsor within the relevant timeframes. The chief investigator shall be responsible for all adverse event reporting.

### Ethics committee and regulatory approval

The trial will be conducted in accordance with ethical principles that have their origin in the Declaration of Helsinki, 1996 [32]; principles of good clinical practice [30], and the Department of Health Research Governance Framework for Health and Social Care, 2005 [33].

### Potential conflicts of interest

The manufacturer of Venofer has had no involvement in design or funding of this study. All drugs are being purchased from the company at normal prices.

## Discussion

Anaemia following hip fracture is common and associated with poorer outcomes for this group of frail patients. However, the optimal treatment for it is not yet known. Blood loss at the time of injury is not amenable to intervention, so therapeutic choices include minimizing further blood loss through surgical, anaesthetic and pharmacological techniques, encouraging haematopoiesis or replacing blood by transfusion. Tranexamic acid, an anti-fibrinolytic agent, has been shown to be beneficial in elective orthopaedic surgery [19,20] and in major trauma. In hip fracture patients, however, it appears to reduce blood loss at the cost of increased cardiovascular morbidity [19]. The impact of blood transfusion on outcomes remains controversial. Some studies support a risk of infective complications from transfusion [15] whereas others have found benefit from transfusion [5,14]. The recently reported FOCUS study found no overall benefit from liberal vs. restrictive transfusion policies [18]. One explanation for these results may be that the risks of blood transfusion broadly balance out the benefits. An alternative approach is therefore to encourage haematopoiesis thereby improving [Hb] without the attendant risks and costs of transfusion. Although the results from the Spanish group are very encouraging, they are not, in themselves, sufficient to recommend routine use of intravenous iron or erythropoietin in this group of patients. The subgroups showing benefit in each of the studies are different, as is the drug protocol. The time to surgery is significantly longer than UK or Scandinavian practice, which may, potentially, have significant impact on perioperative transfusion requirements. We have explicitly defined our subgroups a priori*.* This study is not powered to detect differences in these subgroups, but these subgroups are broadly in line with those where differences in efficacy of i.v. iron have previously been reported. A-priori subgroup analysis may provide suggestive evidence for planning future studies. As with all clinical studies, we have tried to balance pragmatism, generalizability and scientific ‘purity’ in the protocol. We have excluded patients under 70 years of age to reduce the risks of studying a population that is younger and fitter than the ‘average’ hip fracture patient. Our transfusion triggers are a codification of current clinical practice, which broadly reflect UK clinical practice. Inevitably, they will not precisely match practice across the UK or worldwide, but we believe they are similar enough to be applicable elsewhere. On the \basis of current evidence, we do not feel that there is sufficient evidence to either proscribe or demand the use of tranexamic acid. Our local practice is not to delay surgery for patients on clopidogrel or ticlopidine, but they are at increased risk of bleeding in the intra-operative and perioperative period; such patients are therefore excluded from the study. Standard care is similar to most units in the UK, and the randomization process should account for any potential bias due to differences in local practice.

It is unlikely that intravenous iron, as a single intervention, will alter major outcomes, such as length of stay, mortality or readmission rates to a clinically significant degree, but it is plausible for it to have a beneficial effect on transfusion rates. However, this is only likely to happen if a significant effect on erythropoiesis occurs; hence, our choice of the absolute difference in reticulocyte count as the primary outcome of this study. We have chosen day seven as the primary time point for measuring difference in reticulocyte count. Although an effect may be seen at later times, we believe, consistent with the literature [25], that if a clinically significant effect is present, it will be observed within one week. Prolonging the observation time also risks losing participants due to discharge.

If the results of this study are positive, it will provide the necessary information for development of a full-scale trial of intravenous iron. There is an increasing awareness of the benefit of bundles of care [34] as opposed to single ‘magic bullet’ interventions [35] to improve outcomes following surgery. A full-scale trial would help to define the potential cost, resource requirement (blood products) or direct patient benefit of using intravenous iron as part of such a bundle of care. If there is a cost benefit to using intravenous iron, this could be significant: around 80,000 patients have operative fixation of hip fracture in the UK each year [36]. Conversely, if there is no benefit in the UK setting, then healthcare resources could usefully be directed elsewhere.

### Trial status

Approvals were obtained from the Nottingham Research Ethics Committee on 23 November 2011 (reference number 11/EM/0310), the Nottingham University Hospitals Research and Development department on 2 August 2011 (reference number 68213) and the UK Medicines and Healthcare products Regulatory Authority on 19 January 2012, (EuDRACT: 2011-003233-34). The study was also registered with the National Institute for Health Research Portfolio and ISRCTN:76424792 (10 September 2012). The study is ongoing. The first participant was randomized on 19 July 2012; it is hoped that the study will be completed with the last visit of the last patient before 12 March 2014.

## Abbreviations

Hb: Haemoglobin; [Hb]: Concentration of haemoglobin; i.v: Intravenous.

## Competing interests

This study is funded by the National Institute of Academic Anaesthesia and the Association of Anaesthetists of Great Britain and Ireland. The authors do not receive any reimbursement or financial benefits and declare that they have no competing interests.

## Authors’ contributions

IKM is the chief investigator, protocol author and grant holder and drafted and revised the manuscript. OS is an investigator and assisted with the original protocol. DPF and MR are investigators and revised the manuscript. All authors read and approved the final manuscript.
